# G140S/Q148R and N155H mutations render HIV-2 Integrase resistant to Raltegravir whereas Y143C does not

**DOI:** 10.1186/1742-4690-8-68

**Published:** 2011-08-19

**Authors:** Xiao-Ju Ni, Olivier Delelis, Charlotte Charpentier, Alexandre Storto, Gilles Collin, Florence Damond, Diane Descamps, Jean-François Mouscadet

**Affiliations:** 1 LBPA, CNRS, Ecole Normale Supérieure de Cachan, Cachan, France; 2 School of Life Science, East China Normal University, Shanghai, China; 3 Laboratoire de Virologie, AP-HP Groupe Hospitalier Bichat-Claude Bernard, HUPNVS, Université Paris-Diderot, Sorbonne Paris Cité, EA4409, 75018, Paris, France

**Keywords:** HIV-2, integrase, raltegravir, resistance, mutation

## Abstract

**Background:**

HIV-2 is endemic in West Africa and has spread throughout Europe. However, the alternatives for HIV-2-infected patients are more limited than for HIV-1. Raltegravir, an integrase inhibitor, is active against wild-type HIV-2, with a susceptibility to this drug similar to that of HIV-1, and is therefore a promising option for use in the treatment of HIV-2-infected patients. Recent studies have shown that HIV-2 resistance to raltegravir involves one of three resistance mutations, N155H, Q148R/H and Y143C, previously identified as resistance determinants in the HIV-1 integrase coding sequence. The resistance of HIV-1 IN has been confirmed *in vitro *for mutated enzymes harboring these mutations, but no such confirmation has yet been obtained for HIV-2.

**Results:**

The integrase coding sequence was amplified from plasma samples collected from ten patients infected with HIV-2 viruses, of whom three RAL-naïve and seven on RAL-based treatment at the time of virological failure. The genomes of the resistant strains were cloned and three patterns involving N155H, G140S/Q148R or Y143C mutations were identified. Study of the susceptibility of integrases, either amplified from clinical isolates or obtained by mutagenesis demonstrated that mutations at positions 155 and 148 render the integrase resistant to RAL. The G140S mutation conferred little resistance, but compensated for the catalytic defect due to the Q148R mutation. Conversely, Y143C alone did not confer resistance to RAL unless E92Q is also present. Furthermore, the introduction of the Y143C mutation into the N155H resistant background decreased the resistance level of enzymes containing the N155H mutation.

**Conclusion:**

This study confirms that HIV-2 resistance to RAL is due to the N155H, G140S/Q148R or E92Q/Y143C mutations. The N155H and G140S/Q148R mutations make similar contributions to resistance in both HIV-1 and HIV-2, but Y143C is not sufficient to account for the resistance of HIV-2 genomes harboring this mutation. For Y143C to confer resistance *in vitro*, it must be accompanied by E92Q, which therefore plays a more important role in the HIV-2 context than in the HIV-1 context. Finally, the Y143C mutation counteracts the resistance conferred by the N155H mutation, probably accounting for the lack of detection of these mutations together in a single genome.

## Background

HIV-2 is endemic in West Africa and has spread throughout Europe over the last two decades [1,2]. The development of seven different classes of antiretroviral drugs has led to the establishment of highly active treatments that have had a profound effect on the morbidity and mortality of HIV-1-infected individuals. These classes are nucleoside (NRTIs), nucleotide (NtRTIs) and non nucleoside (NNRTIs) reverse transcriptase inhibitors, protease inhibitors (PIs), entry inhibitors, fusion inhibitors and integrase (IN) inhibitors (INIs). Despite this apparent diversity, the alternatives for HIV-2-infected patients are more limited because NNRTIs and fusion inhibitors are not active against HIV-2 [3,4] and HIV-2 is also less sensitive to some PIs [5-7]. It has also been suggested that the genetic barrier is weaker in HIV-2, potentially resulting in the more rapid emergence of resistance to other PIs [8,9]. The development of novel treatments based on drug classes highly effective against HIV-2 is therefore essential. INIs are active against HIV-2 IN and are therefore a promising option for use in the treatment of HIV-2-infected patients [10,11]. IN plays a key role in the viral replication cycle. This makes it an attractive target for antiretroviral therapy, together with two other enzymes: reverse transcriptase (RT) and protease (P). The viral integrase catalyzes two spatially and temporally independent reactions, which eventually lead to covalent insertion of the viral genome into the chromosomal DNA. The first reaction, 3'-processing, is an endonucleolytic cleavage trimming both the 3'-extremities of the viral DNA, whereas the second reaction, strand transfer, results in the concomitant insertion of both ends of the viral DNA into a host-cell chromosome through one-step transesterification. IN strand transfer inhibitors (INSTIs) are specific inhibitors of the strand transfer reaction. The flagship molecule in this class is raltegravir (RAL), the first INSTI to have received approval for clinical use for both treatment-experienced and treatment-naïve patients [12]. RAL has a rapid and sustained antiretroviral effect in patients with advanced HIV-1 infection [13,14]. As it has a different mechanism of action, RAL is also effective against viruses resistant to other classes of antiretroviral drugs [13]. Moreover, although HIV-1 and HIV-2 IN nucleotide sequences are only 40% identical, RAL is active against wild-type HIV-2, which has a phenotypic susceptibility to this drug similar to that of HIV-1 [11,15].

However, as for other antiviral drugs, resistance to RAL emerges rapidly both *in vitro *and *in vivo*, through the selection of mutations within the IN coding region of the *pol *gene, greatly reducing the susceptibility of the virus to the inhibitor. In HIV-1, three main resistance pathways, involving the residues N155, Q148 and Y143, have been shown to confer resistance to RAL *in vivo*. The virological failure of RAL-based treatment in HIV-1 infection is associated primarily with the initial, independent development of the principal N155H and Q148H/K/R pathways, either alone or together with other resistance mutations. Secondary resistance mutations, such as G140S, which have little or no direct effect on drug susceptibility *per se*, increase phenotypic resistance or viral fitness [16]. More than 60 mutations have been shown to be specifically associated with resistance to INSTIs, but biochemical studies have demonstrated that the mutations affecting residues Y143, Q148 and N155 are sufficient to decrease the susceptibility of IN to the inhibitor *in vitro *[16-18]. The third pathway, involving the Y143R/C mutation, is less frequently observed and was identified after the N155 and Q148 pathways [17,19,20].

Recent phenotypic studies have established that HIV-2 resistance to RAL may also involve one of the three primary resistance mutations: N155, Q148 and Y143 [10,21,22]. However, whereas the resistance of HIV-1 IN to RAL has been confirmed *in vitro *with IN site-directed mutants harboring these mutations, no such study has yet been carried out for the HIV-2 proteins [16,17,23]. We describe here the *in vitro *catalytic activity and resistance to RAL of HIV-2 recombinant IN isolated from clinical isolates harboring resistance mutations. By comparing these isolates with IN mutants generated by single-site mutagenesis, we demonstrate that G140S/Q148R and N155H are sufficient to confer resistance to RAL, whereas Y143C mutation is not. We show also that N155H and Y143C mutations have antagonistic effects.

## Results

### Analysis of HIV-2 IN sequences from clinical isolates before RAL-based treatment

The complete sequence of the HIV-2 IN coding region from clinical isolates N1 to N3 was determined by amplification, cloning and sequencing of the IN coding region of the *pol *gene from plasma samples obtained at the start of RAL-based treatment. All three isolates were HIV-2 group B, as shown by comparisons with the HIV-2 group B reference sequence EHO, from which they diverged very little (between 3% and 5% over the first 293 residues) (Table 1). Substitutions with respect to the HIV-2 EHO sequence were found in all three viruses, at nine residues (N17, R34, I133, T180, T215, R224, N270, M287, V292) and in one or two viruses at eight other residues (F26, I50, D125, D163, V175, I204, Q221, I260). These results are consistent with previous estimates of variation for group B isolates [11]. The divergence between the three isolates was even weaker, with only ten residues displaying variation, mostly conservative, in one of the three sequences (F26, I50, D125, D163, V175, T180, I204, T215, Q221, I260), demonstrating a high degree of conservation of the IN sequence (Table 1). None of these substitutions affected a residue previously associated with INSTI resistance *in vivo*, consistent with the absence of prior exposure to INIs. As expected for group B sequences, the C-terminal domain was of variable length. Thus, the full length of IN was 301 codons for the virus from patient N1, 299 for the virus from patient N2 and 293 for the virus from patient N3. The N1 and N2 sequences had an AQS motif for codons 293 to 295, consistent with the EHO/B reference sequence.

**Table 1:** Amino acid variations of HIV-2 IN isolates from INI-naive patients

		EHO Reference sequence																
**Patient**	**Group**	**17**	**26**	**34**	**50**	**125**	**133**	**163**	**175**	**180**	**204**	**215**	**221**	**224**	**260**	**270**	**287**	**292**
		**N**	**F**	**R**	**I**	**D**	**I**	**D**	**V**	**T**	**I**	**T**	**Q**	**R**	**I**	**N**	**M**	**V**

N1	B	G	-	K	V	-	V	-	-	V	V	A	-	Q	-	H	R	M
N2	B	G	-	K	-	-	V	-	I	A	-	N	-	Q	V	H	R	M
N3	B	G	Y	K	V	E	V	N	-	A	-	A	K	Q	V	H	R	M

**Figure 1:**
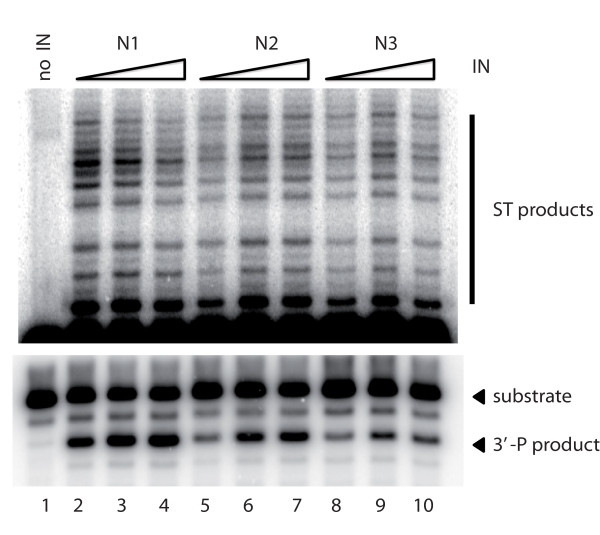
**Study of catalytic properties of INs amplified from plasma of three INI-naive patients (N1, N2, N3) infected with HIV-2**. Processing activity (bottom panel) and strand transfer activity (top panel) were assayed as a function of IN concentration. Both panels represent a unique gel with the top panel corresponding to a longer exposure. Full assay for catalytic activity was performed as described in Materials and methods section using a 21-mer (U5A/U5B) blunt DNA substrate (12,5 nM), with MgCl2 as a cofactor (7.5 mM). Lane 1. No IN. Lanes 2, 5, 8: 100 nM; Lanes 3, 6, 9: 200 nM; Lanes 4, 7, 10: 300 nM IN. ST: strand transfer; 3'-P: 3'-processing.

We investigated possible effects of sequence and length variation on IN activity, by producing and purifying the three proteins according to the protocol developed for HIV-1 IN, which favors the physiological Mg^2+^-dependent activity of the protein [24]. The three enzymes, N1, N2 and N3, performed both catalytic activities efficiently (Figure 1). Differences in specific activity were observed, through assessments of the amount of product obtained as a function of enzyme concentration, but they remained within the range of variation for recombinant IN preparations, suggesting that neither divergence at the C-terminal end nor sequence variation had a significant impact on enzyme activity *in vitro*.

**Figure 2:**
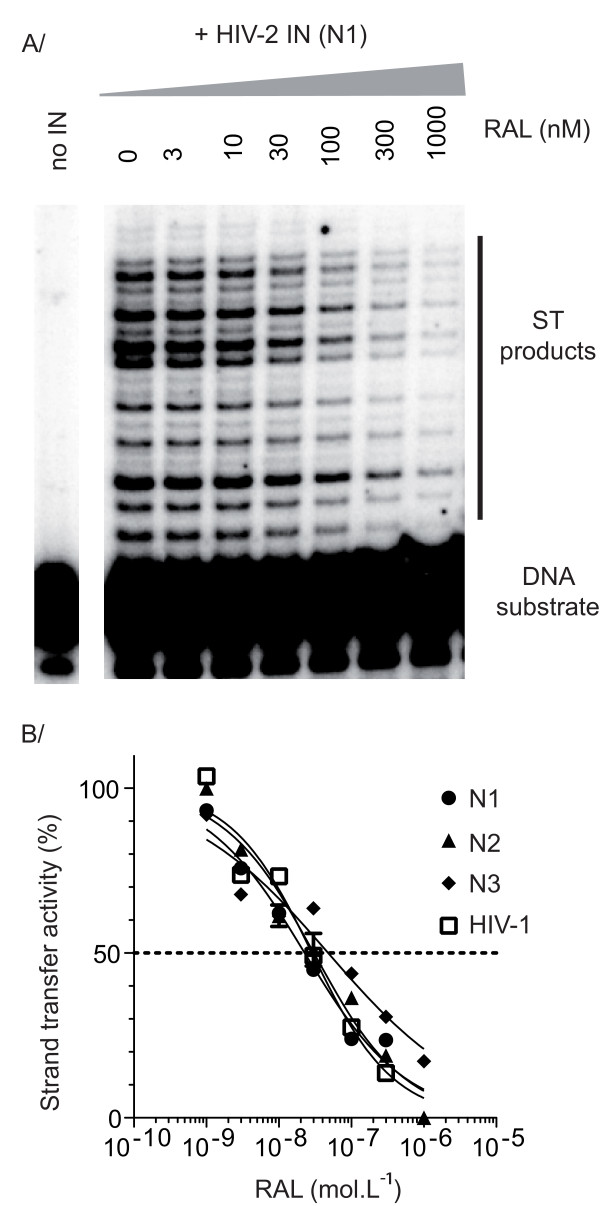
**Comparison of in vitro RAL susceptibility of HIV-2 N1-N3 and HIV-1 HxB2 INs**. (A) A representative gel obtained for HIV-2 N1 IN-mediated strand transfer reaction in the presence of increasing concentrations of raltegravir. The ST reaction was performed using a ^32^P-labeled oligonucleotide mimicking the preprocessed substrate. Drug concentrations are indicated above each lane. (B) Susceptibility to RAL of HIV-2 INs. Strand transfer reaction was carried out for three hours in the presence of 200 nM IN and increasing concentrations of RAL. Activity is expressed as a % of control without drug. Experiments were repeated two times.

The enzymes from clinical isolates N1 to N3 were obtained from plasma samples collected before treatment with RAL. We confirmed the susceptibility of these INs to this INSTI, by determining IC_50 _values *in vitro *in dose-response assays carried out in the presence of various concentrations of inhibitor. RAL efficiently inhibited the strand transfer activity of the enzyme (Figure 2A), but had no significant effect on 3' processing *in vitro *at concentrations up to 1 μM (data not shown), as expected for an INSTI. All three enzymes were susceptible to RAL (Figure 2B). The clinical isolates of HIV-2 studied were, therefore, susceptible to RAL before treatment initiation. The IC_50 _values obtained for enzymes from clinical isolates N1 and N2 were respectively equal to 23 nM and 30 nM, similar to that obtained for HxB2 HIV-1 IN (IC_50 _= 28 nM) in these experimental conditions, whereas the IN from N3 had a slightly lower susceptibility to RAL (IC_50 _= 48 nM). Thus, sequence polymorphism may slightly affect IN susceptibility to RAL.

### Identification of mutations associated with RAL resistance

Plasma samples were collected from seven antiretroviral-experienced RAL-treated HIV-2-infected patients (patients T1 to T7), at the time of RAL treatment failure. Complete IN sequences were obtained by amplification, cloning and sequencing of the IN coding region. Patients T1 and T2 were infected with HIV-2 group B and patients T3 to T7 were infected with HIV-2 group A (Table 2). Comparison with the HIV-2 group A reference sequence ROD showed that all five group A sequences displayed the following polymorphisms V19I, R34K, S39T, A41T, T60V, I133V, I180V, I201T, E207D at positions that were previously showed to be subject to variation [11]. All group A viruses harbored a Q148R resistance mutation, associated with G140S in two cases (patients T3 and T4) and G140A in two others (patients T5 and T7; Table 2).

**Table 2:** Amino acid substitutions of HIV-2 IN at RAL failure

Sequence	Residue			
	**92**	**97**	**143**	**155**

EHO (B)	E	T	Y	N
Pat. T1	A	A	-	H
Pat. T2	Q	-	C	-
		
	140	148		
		
ROD (A)	G	Q		
Pat. T3	S	R		
Pat. T4	S	R		
Pat.T5	A	R		
Pat. T6	-	R		
Pat. T7	A	R		

The two B viruses differed from the EHO reference sequence by identical variations at the following residues: N17G, R34K, S56A, V72I, I84V, A129V, I133V, E146Q, T180V, I201L, L213F, T215A, R224Q, D240E and N270H. One group B sequence harbored the N155H resistance mutation (patient T1), and another had the Y143C resistance mutation (patient T2) (Table 2). The N155H-mutated virus also harbored the E92A and T97A mutations known to be associated with RAL resistance. Several substitutions, G27E, G70E, G82R, E92Q, and Q124R, were also detected in the Y143C-mutated virus, including one (E92Q) known to be associated with RAL resistance. These data confirm that the three main mutation patterns giving rise to RAL resistance in HIV-1 are also associated with resistance in HIV-2, as suggested by the direct sequencing of viral genomes in plasma samples [15,21].

### *In vitro *enzymatic activity of HIV-2 Ins

**Figure 3:**
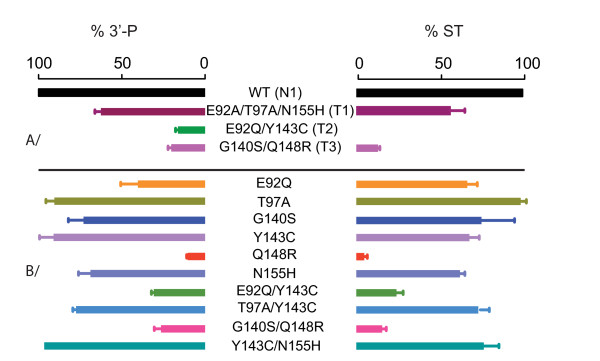
***In vitro *enzymatic activity of HIV-2 INs**. 3'-processing (3'-P) and strand stranfer (ST) activities of the mutants are normalized and represented as percentage of activity wild-type B-type sequence of INI-naïve patient N1 that was taken as a reference for HIV-2 IN activity. A/INs amplified from HIV-2 infected patients. B/INs harboring mutations obtained by site-directed mutagenesis in N1 background.

Biochemical studies have demonstrated that Q148R, N155H and Y143C are primary resistance mutations giving rise to HIV-1 resistance whereas G140S/A and E92Q are secondary resistance mutations increasing resistance and viral fitness [16,17]. We determined whether the roles of these mutations were similar in HIV-2, by producing and purifying three recombinant IN sequences corresponding to clinical isolates T1 (N155H; R1), T2 (Y143C; R2) and T3 (G140S/Q148R; R3). The catalytic activities of these enzymes were assessed and compared with that of the wild-type susceptible enzyme from patient N1, used as a control (Figure 3). Both catalytic activities were affected, to various extents, in all three enzymes. The N155H-containing enzyme displayed about 60% the activity of the control *in vitro*, within the range of variation for wild-type enzymes. The G140S/Q148R double mutant was more strongly impaired, displaying only 30% control levels of activity, and E92Q/Y143C mutant activity was barely detectable under standard conditions *in vitro*, indicating a functional defect.

**Figure 4:**
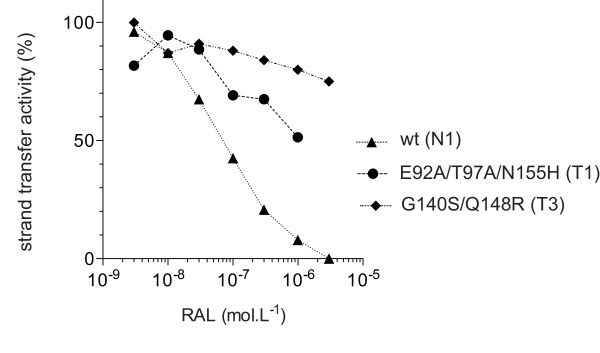
***In vitro *RAL susceptibility of the HIV-2 reference (N1) and T1 and T3 resistant INs amplified form clinical isolates**. Strand transfer reaction was carried using a 32P-labeled oligonucleotide mimicking the preprocessed substrate and 200 nM IN, in the presence of increasing concentrations of RAL at 37°C. Activity is expressed as a % of control without drug. Experiments were performed two times.

The mutant enzymes harboring the E92A/T97A/N155H (T1) and G140S/Q148R (T3) mutations retained sufficient strand transfer activity for tests of their susceptibility to RAL, whereas this was not the case for enzymes with E92Q/Y143C (T2) mutations. The strand transfer activity of HIV-2 IN was measured in the presence of various concentrations of RAL (Figure 4). N155H mutation, in conjunction with secondary substitutions at positions 92 and 97, increased the IC_50 _by a factor of about 50, whereas the IC_50 _was not reached for the G140S/Q148R double mutant, for concentrations up to 1 μM. Thus both the N155H- and G140S/Q148R-containing enzymes were much less susceptible to RAL *in vitro *than the wild-type N1 HIV-2 IN, confirming that these mutations were the cause of viral resistance to RAL.

### Effect of single and double mutations on IN activity *in vitro*

**Figure 5:**
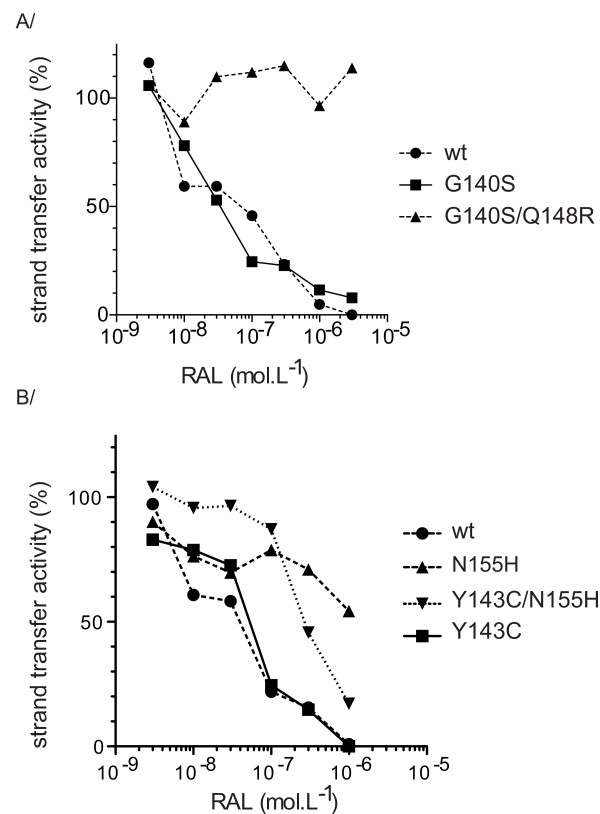
***In vitro *RAL susceptibility of wt and mutated HIV-2 INs**. Mutations were introduced in the HIV-2 N1 background by mutagenesis. (A) Comparison of strand transfer activity in the presence of RAL of wt (circle), G140S (square) and G140S/Q148R (triangle) mutants. (B) Comparison of strand transfer activity in the presence of RAL of wt (circle), N155H (triangle), Y143C (square) and N155H/Y143C (inverted triangle) HIV-2 INs. Strand transfer reaction was carried using a 32^P^-labeled oligonucleotide mimicking the preprocessed substrate and 200 nM IN, in the presence of increasing concentrations of RAL at 37°C. Activity is expressed as a % of control without drug. Experiments were performed two times.

We investigated the contribution of each individual mutation to RAL resistance, by introducing G140S, Q148R, N155H and Y143C single mutations and the G140S/Q148R double mutation into the HIV-2 wild-type IN N1 sequence by site-directed mutagenesis. We first assessed the impact of these mutations on enzymatic activity *in vitro*, for both the 3'-processing and strand transfer activities, by comparing the efficiency of IN activities with that of the wild-type reference N1 enzyme. HIV-2 IN harboring the mutation Q148R had a much lower level of catalytic activity (<10% wild-type levels) than the wild-type enzyme (Figure 3). By contrast, the N155H mutation had no significant effect on IN activity. Introduction of the secondary mutation G140S into the Q148R background resulted in the partial recovery (up to 30% of wild-type levels) of IN catalytic activity, which was strongly impaired by the Q148R mutation. This result is similar to that obtained for HIV-1 [16]. The recombinant enzymes harboring the N155H, Y143C, G140S and G140S/Q148R mutations were assayed for susceptibility to RAL. The Q148R-containing enzyme only had low levels of activity precluding precise evaluation of its resistance but preliminary studies with high protein concentrations suggested that this enzyme was not susceptible to RAL. The G140S mutant retained full activity and was as susceptible to RAL as the wild-type reference N1 enzyme (Figure 5A). By contrast, introduction of the G140S mutation into the Q148R background yielded a protein that was highly resistant to RAL. Thus, the G140S and Q148R mutations play the same role in the resistance of IN to RAL as in the HIV-1 integrase. Introduction of the N155H mutation into the wild-type background also resulted in a high level of resistance (Figure 5B), with a fold-change with respect to the wild-type enzyme similar to that for the clinical isolate harboring the E92A/T97A/N155H triple mutation, which confirmed the identification of N155H as a primary resistance mutation for HIV-1 IN [22]. By contrast, introduction of the Y143C mutation did not lead to significant resistance of the protein *in vitro*, suggesting that Y143C is not a primary RAL resistance mutation in HIV-2 IN.

Population sequencing results suggested that resistant viruses could harbor both the Y143C and 155H mutations. We tested this hypothesis by assaying the double mutant. However, introduction of the Y143C mutation into an N155H background significantly decreased resistance levels, by one order of magnitude (IC_50 _= 290 nM) with respect to the resistant N155H-containing IN (Figure 5B). The solubility of the Y143C/N155H recombinant IN was also lower than that of the other HIV-2 INs, suggesting that proteins carrying both these mutations were unable to adopt an appropriate conformation. Consistent with this hypothesis, the catalytic activity of the Y143C/N155H recombinant mutant was strongly increased by the addition of 10% DMSO (data not shown), probably because DMSO may help to stabilize partially folded conformations of proteins [25]. These observations suggest that the Y143C and N155H mutations are mutually exclusive in a context of natural selection.

**Figure 6:**
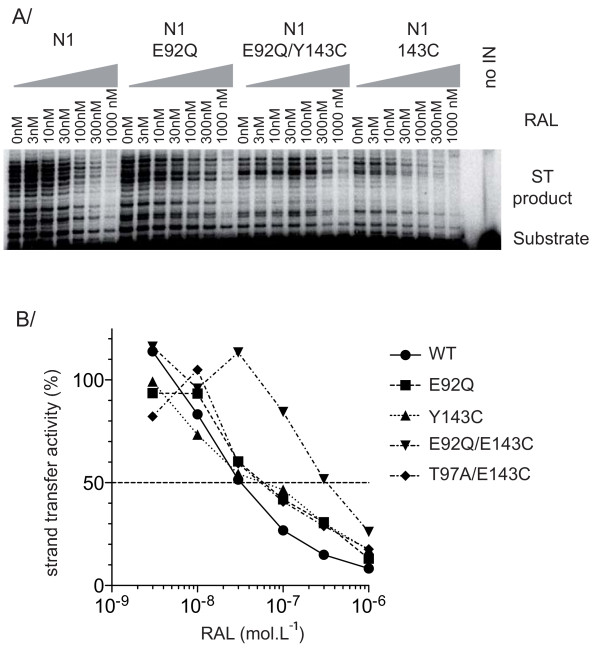
***In vitro *RAL susceptibility of wt and mutated HIV-2 INs**. Mutations were introduced in the HIV-2 N1 background by mutagenesis. (A) Representative gel of the strand transfer activity of recombinant HIV-2 IN mutants in the presence of increasing concentrations of RAL. (B) Comparison of strand transfer activity in the presence of RAL of wt (N1) (circle), E92Q (square), Y143C (triangle), E92Q/Y143C (inverted triangle) and T97A/Y143C (diamond) mutants. Strand transfer reaction was carried using a ^32^P-labeled oligonucleotide mimicking the preprocessed substrate and 200 nM IN, in the presence of increasing concentrations of RAL at 37°C. Activity is expressed as a % of control without drug. Experiments were performed two times.

The two group B recombinant enzymes amplified from clinical isolates T1 and T2 at the time of virological failure contained E92A/Q mutations. Such mutations were previously implicated in the resistance of HIV-1 IN to INSTIs. We investigated the role of the E92 mutation by preparing the E92Q single mutant and the E92Q/Y143C double mutant in the wild-type N1 background. Both enzymes were active *in vitro*, suggesting that the impairment of the catalytic activity of the T2 IN (E92Q/Y143C) was not directly related to the presence of these mutations (Figure 6A). The susceptibility of recombinant INs to RAL was determined by quantifying the inhibition of *in vitro *strand transfer activity in the presence of various concentrations of RAL (Figure 6B). Like Y143C, E92Q alone did not confer significant resistance to RAL *in vitro*. By contrast, the resistance level increased when E92Q was introduced into a Y143C resistant background (IC_50 _= 370 nM), suggesting that the concomitant presence of the two mutations was necessary for this significant increase in resistance.

Finally, it has been suggested that the T97A mutation is involved in resistance to RAL. We tested this hypothesis, by producing a T97A-containing IN. The introduction of T97A into the Y143C background did not lead to detectable IN resistance *in vitro *(Figure 6B), ruling out a direct role for this mutation in RAL resistance. Nevertheless, IN harboring the T97A substitution has higher levels of activity, suggesting that T97A may increase the fitness of resistant viral mutants.

## Discussion

RAL has been reported to be clinically effective against HIV-2 infection [26,27]. However, HIV-1 and HIV-2 IN nucleotide sequences are only 40% identical, and their amino acid sequences are only 65% similar, suggesting possible differences in the response of the enzymes to inhibitors and in the mechanisms by which resistance emerges. In this study, we characterized the *in vitro *activity of HIV-2 IN produced from the IN coding region amplified from three plasma samples taken from RAL naive, HIV-2-infected patients, and from seven others after virological failure following exposure to RAL.

Clonal analysis of three IN sequences recovered from the untreated patients identified HIV-2 group B sequences with different length as already observed for groups A and B HIV-2 IN [11]. The three IN sequences studied all displayed the N17G, R34K, I133V, R224Q, N270H, M287R and V292M variations, with respect to the EHO group B reference sequence, suggesting that these variations are prevalent and may even form a consensus in HIV-2 group B circulating strains. This is particularly likely for the G17, V133 and Q224 residues, which were also detected in all five group A sequences amplified. The residues involved in resistance are therefore probably less variable than previously thought [11].

The catalytic activities of the three proteins were similar, and the specific activities of the enzymes *in vitro *were similar to that of an HIV-1 IN control, indicating a lack of impact of C-terminal variation on enzyme activity. HIV-2 IN-mediated strand transfer was inhibited to the same extent by RAL *in vitro *as HIV-1 IN strand transfer, consistent with the similar phenotypic susceptibilities of HIV-1 and HIV-2 to this class of inhibitor. This result also indicates that RAL interacts with these two INs in similar ways. This conclusion is strongly supported by the complete conservation in HIV-2 of the HIV-1 IN residues C65, T66, H67, N117, F121, T122, A128, G149 and K159, which have been implicated in the interaction with RAL [28].

Whole-population analysis of viral samples recovered from HIV-2-infected patients who experienced virological failure on RAL treatment indicated that the three main pathways of RAL resistance in HIV-1, involving mutations of residues Q148, N155 and Y143, may be also responsible for the resistance of HIV-2 to INSTIs [26,29,30]. Moreover, a previous clonal analysis found that E92Q was associated with both the N155H and Y143C mutations, whereas T97A mutation was associated with Y143C [15]. Our clonal analysis based on seven clinical isolates identified three different patterns, E92A/T97A/N155H, G140S/Q148R and E92Q/143C, confirming the probable existence of these three pathways.

The three recombinant INs obtained by cloning and expressing these sequences were indeed strongly resistant to RAL *in vitro*, thereby confirming that the mutated sequence was responsible for resistance. We showed, by single-site mutagenesis in a RAL-susceptible background, that the N155H mutation and the G140S/Q148H double mutation were sufficient to elicit strong resistance to RAL in HIV-2 IN *in vitro*. For the G140S/Q148H pattern, Q148H caused a major catalytic defect while conferring a high level of resistance to RAL, the catalytic defect being rescued *in vitro *by the secondary G140S mutation, which did not itself confer resistance. This result is entirely consistent with our previous observations for HIV-1 *in vitro *[16]. The concomitant selection of the Q148H/R/K and G140S mutations in both HIV-1 and HIV-2 RAL-resistant viruses is grounded in the structure of IN, indicating a close interaction between these two residues that are conserved in retroviral IN [31].

A whole-population study suggested a possible association of T97A with both N155H and Y143C mutations in resistant HIV-2 viruses [21,32]. For the N155H mutation, the level of resistance conferred by single-site directed mutagenesis was very similar to that of the enzyme amplified from the E92A/T97A/N155H clinical isolate, suggesting that the E92A and T97A secondary mutations provided no additional resistance. This hypothesis is consistent with the absence of resistance observed for INs into which T97A and E92Q were introduced as single mutations. Thus, N155H is the main, if not only determinant of viral resistance in this pathway, consistent with the findings of previous phenotypic studies of viral replication [22]. Similarly, we observed no significant effect of T97A in combination with Y143C, on HIV-2 IN susceptibility to RAL. Nevertheless, limited stimulation of IN activity was detected, suggesting that this mutation may improve the fitness of enzymes that would otherwise be catalytically impaired as previously suggested for HIV-1 IN [33].

Y143C/R has been described as a primary mutation for HIV-1 resistance to RAL [19]. We previously demonstrated *in vitro *that the susceptibility to RAL of IN was strongly affected by this single mutation [17]. Surprisingly, we observed that, although the Y143C-containing HIV-2 IN was amplified from clinical isolate of a patient at time of RAL failure, Y143C mutation alone was not sufficient to confer resistance to IN *in vitro*, ruling out this mutation as a sole determinant of resistance in the HIV-2 context. The IN sequence amplified from the clinical isolate also contained the E92Q mutation. We therefore studied the impact of this mutation within the Y143C background. E92Q is considered to be a secondary mutation within the N155H and Y143C pathways for HIV-1 resistance to RAL. Indeed, E92Q confers resistance to HIV-1 IN *in vitro*, albeit to a lesser extent than N155H or Q148R [18]. However, this effect was not confirmed in the HIV-2 context, because the E92Q mutation, introduced through single-site directed mutagenesis, did not significantly increase the resistance of the HIV-2 mutant IN *in vitro*. Nonetheless, the Y143C/E92Q double mutation obtained by mutagenesis of the wild-type control resulted in resistance *in vitro*. Thus, although neither Y143C nor E92Q is sufficient for significant resistance to RAL, the presence of both these substitutions in the same protein leads to resistance indicating that unlike HIV-1, at least two mutations seem to be required in this pathway to elicit resistance in HIV-2. A recent structural study suggested that RAL establishes contact with the side chain of Y143 [31]. We conclude that, in HIV-2, the loss of this contact is not sufficient to impair RAL binding to IN. A second modification to the RAL binding site, which encompasses E92, is probably required. E92Q has been described as a primary resistance mutation for another INSTI, elvitegravir. Moreover, it was also shown that elvitegravir remains fully active against Y143 mutant HIV-1 integrase [34]. It would therefore be interesting to determine whether there are structural similarities between elvitegravir binding to HIV-1 IN and RAL binding to HIV-2 IN.

It has been suggested that the emergence of Y143C during HIV-1 infection may result from a late switch from the N155H pathway [19]. Our data show that, in HIV-2 infection, the Y143C mutation counteracts the resistance conferred by the N155H mutation, probably precluding the simultaneous selection of both mutations. Thus, although we did not investigate the effect of E92 mutations on the *in vitro *resistance of N155H-containing HIV-2 IN, we suggest that the emergence of E92A/G/Q secondary mutations, facilitated by the single nucleotide change required for all substitutions -- E to A, E to G or E to Q transition -- may be involved in the switch from the N155H to the Y143C pathway in the HIV-2 context. Under this hypothesis, Y143C-containing resistant viruses would be expected to be more resistant to RAL than N155H-containing viruses. This was not the case here, as the Y143C/E92Q recombinant enzyme was less resistant than the N155H-containing enzyme. There may therefore be other, as yet unidentified determinants. Y143C/E92Q-containing INs from clinical isolates also harbored G27E, G70E, G82R and Q124R variants, which were not found in the HIV-2 group B RAL-susceptible sequences. Moreover, none of these residues has previously been identified as a site of major variation, consistent with selection under RAL pressure in these isolates. However, these residues have also never before been associated with the resistance of HIV-1 or HIV-2 to INSTIs, and their role remains to be determined [35,36].

## Conclusion

In conclusion, this study confirms that HIV-2 resistance to RAL is due to the N155H, G140S/Q148R or E92Q/Y143C mutations in the IN coding region. The N155H and G140S/Q148R mutations make similar contributions to resistance in both HIV-1 and HIV-2, but Y143C alone is not sufficient to account for the resistance of HIV-2 genomes harboring this mutation. For Y143C to confer resistance in vitro, it must be accompanied by E92Q, which therefore plays a more important role in the HIV-2 context than in the HIV-1 context.

## Methods

### Patients

Plasma samples were collected from three RAL-naive patients infected with HIV-2, group B (patients N1 to N3). Two of these patients had previously received antiretroviral treatment whereas the third had never been treated. Plasma samples were also collected from seven different HIV-2-infected patients, two group B and five group A, at the time of virological failure on RAL-based treatment (patients T1 to T7).

### Cloning and site-directed mutagenesis

The IN coding sequences from the viruses isolated from the plasma of HIV-2-infected patients N1 to N3 and T1 to T7 were amplified by PCR and sequenced according to a previously described procedure [11]. PCR products corresponding to the entire IN sequence were amplified again with the following specific primers containing *Nde*I and *Bam*H I restriction sites at their 5' ends. Primer 1: N1, N2, T1, T2, 5'-CATATGTTTCTAGAAAAGATAGAACCAGC-3'; N3, 5'-CATATGTTTTTAGAG AACATAGAACCAGC-3'; T3-T7, 5'-CATATGTTCCTGGAAAAGATAGAGCCCGC-3'. Primers 2: N1, 5'-GGATCCCTATGCTTCAGGTACTTGACCAG-3'; N2, 5'-GGATCCCAT CCTGGTATCCTCCACGTCGGC-3'; N3, 5'-GGATCCCTATGCCACCTCTCTAGTCTGC C-3'; T1, T2, 5'-GGATCCTTAATTAGACTGTGCCACCTCTCTAG-3'; T3-T7, 5'-GGATC CCTATGCCACCTCTCCATCCTCCCTG -3'. The amplicons were then ligated into the TA cloning plasmid pGEM-T easy (Promega, Madison, USA). Single (E92Q, T97A, G140S, Q148R, Y143C, N155H) and double (G140S/Q148R, Y143C/N155H, E92Q/Y143C and T97A/Y143C) mutations were introduced into the HIV-2 wild-type N1 sequence by mutagenesis using the QuickChange II site-directed mutagenesis kit (Agilent Technologies, Santa Clara, USA) according to the manufacturer instructions. *Nde*I - *Bam*H fragments covering the IN coding sequence were then inserted into the expression vector pET-15b.

### Expression and purification of recombinant Ins

His-tagged INs were produced in *Escherichia coli *BL21-CodonPlus (DE3)-RIPL (Agilent, Santa Clara, USA) and purified under nondenaturing conditions, as previously described [24]. Protein production was induced at an OD_600 _of 0.6 to 0.8, by adding isopropyl β-D-1-thiogalactopyranoside (IPTG) to a concentration of 0.5 mM. Cultures were incubated for 3 h at 30°C and then centrifuged 20 min at 1100 *g*, 4°C. Cells were resuspended in buffer A (50 mM Tris-HCl (pH 8), 1 M NaCl, 4 mM β-mercaptoethanol) and lysed by passage through a French press. The lysate was centrifuged (30 min at 12,000 g, 4°C), and the supernatant was filtered (pore size 0.45 μM) and incubated with nickel-nitrilotriacetic acid agarose beads (Qiagen, Venlo, The Netherlands) for at least 2 hours at 4°C. The beads were washed with buffer A and then with buffer A supplemented with 80 mM imidazole. His-tagged proteins were then eluted from the beads in buffer A supplemented with 1 M imidazole and 50 μM zinc sulfate. They were then dialyzed overnight against 20 mM Tris-HCl (pH 8), 1 M NaCl, 4 mM β-mercaptoethanol and 10% glycerol. Aliquots of the purification products were rapidly frozen and stored at -80°C.

### Characterization of IN enzymatic activity *in vitro*

The activity of wild-type and mutated INs was determined *in vitro*, as previously described [24]. Briefly, oligonucleotides (ODN) mimicking the end of the U5 long terminal repeat of the viral genome were radiolabeled with T4 polynucleotide kinase (Biolabs, Ipswich, USA) and [γ-^32^P] ATP (3,000 Ci/mmol) (Amersham, GE Healthcare, USA), then purified on a Sephadex G-10 column (GE Healthcare, USA). Double-stranded ODNs were obtained by mixing equimolar amounts of complementary strands in the presence of 100 mM NaCl. We carried out 3'-processing and strand transfer assays at 37°C in a buffer containing 20 mM HEPES (pH 6.8), 1 mM dithiothreitol (DTT), 7.5 mM Mg^2+ ^and 50 mM NaCl in the presence of a 12.5 nM solution of U5A/U5B (3'-processing) or U5A/U5B-2 (strand transfer) double-stranded DNA substrates, respectively. The products were separated by electrophoresis in a 16% acrylamide/urea denaturing gel. Gels were analyzed with a Typhoon TRIO variable mode imager (GE Healthcare, USA) and quantified with ImageQuant TL software. The susceptibility of INs to RAL was determined *in vitro *by assessing IN activity in the presence of various concentrations of RAL. We obtained 50% inhibitory concentrations (IC_50_) with Prism 5.0 software (GraphPad Software, San Diego, CA). The HIV-2 ODN substrate sequences were: UA: 5'-CCTGCTAGGGATTTTCCTGCCTCGGTTT-3'; U5B: 5'-AAACCGAGGCAGGAAAATCCCTAGCAGG-3'; U5B-2: 5'-AAACCGAGGCAGGAAAA TCCCTAGCA-3'.

## Competing interests

The authors declare that they have no competing interests.

## Authors' contributions

XN carried out the cloning, expression and purification of recombinant integrases, performed the *in vitro *activity studies and contributed to the writing of the manuscript, OD participated in the design of the study and in the enzyme mutagenesis, CC carried out the sequence alignment, contributed to the data interpretation and participated in the writing of the manuscript, AS, GC and FD contributed to the sequence amplifications, analysis and interpretation of the genetic data, DD participated to the conception of the study and to the data interpretation, JFM conceived the study and wrote the manuscript. All authors contributed to the critical reviewing of the manuscript and approved the final manuscript.
